# Supplementary material for: An Overview of the Potential of Food-Based Carbon Dots for Biomedical Applications
Source: Int J Mol Sci. 2023 Nov 21;24(23):16579. doi: 10.3390/ijms242316579 (PMC10706188; doi:10.3390/ijms242316579)
Supplement: Supplementary file 1 [file ijms-24-16579-s001.zip › ijms-2672132-supplementary.docx]

***Supporting Information***

**An Overview of the Potential of Food-Based Carbon Dots for Biomedical Applications**

Chen-Yow Wang ^1^, Nodali Ndraha ^1,2^, Ren-Siang Wu ^3^, Hsin-Yun Liu ^1^, Sin-Wei Lin ^1^, Kuang-Min Yang ^1^ and Hung-Yun Lin ^4,^*

^1^ Department of Bioscience and Biotechnology, National Taiwan Ocean University,
Keelung 202301, Taiwan; 101127xd@gmail.com (C.-Y.W.); nodali@email.ntou.edu.tw (N.N.); tiggermss818@gmail.com (H.-Y.L.); andy920140@gmail.com (S.-W.L.);
ab0913669898@gmail.com (K.-M.Y.)

^2^ Department of Food Science, National Taiwan Ocean University, Keelung 202301, Taiwan

^3^ Division of Microbiology and Immunology, Graduate Institute of Biomedical Sciences, College of Medicine, Chang Gung University, Taoyuan 333323, Taiwan; jerry0127people2017@gmail.com

^4^ Center of Excellence for the Oceans, National Taiwan Ocean University, Keelung 202301, Taiwan

* Correspondence: hungyun@mail.ntou.edu.tw

**Table of contents:**

**Table S1.** Food additive applications for dietary compounds.

**Table S2.** The function of Food additive agents to normal conditions.

**Table S1. Food additive applications for dietary compounds.**

| Compounds | Food Additive Applications |
| --- | --- |
| Alginate | Thickening agent |
| Ammonium citrate | Acidity regulator |
| Ammonium sulfate | Acidity regulator |
| Boronic acid | Preservative agent |
| Carrageenan | Preservative agent and thickening agent |
| Citric acid | Nutritive sour flavoring agent |
| Chitosan | Preservative agent, finings agent, emulsifying agent, and texture agent |
| Diethylenetriamine | Chelating agent |
| Ethylenediamine | Emulsifying agent and chelating agent |
| Folic acid | Vitamin supplement |
| Fucoidan | Food antioxidant and thickening agent |
| Glucose | Nutritive sweetener |
| Glutathione | Food antioxidant |
| Glycyrrhizic acid | Nutritive sweetener |
| Hesperidin | Food antioxidant |
| Microcrystalline cellulose | Anticaking agent, emulsifying agent, foaming agents, and thickener |
| Polyethyleneimine (PEI) | Fixing agent |
| Polyethylene glycol (PEG) | Emulsifying agent |
| Pullulan | Thickening agent |
| Quercetin | Food antioxidant |
| Sorbitan monolaurate | Emulsifying agent and stabilizer |
| Tripolyphosphate | Water retention agent |
| Vit C | Food antioxidant |

**Table S2. The function of Food additive agents.**

| Food Additive Agents | Function |
| --- | --- |
| Acidity regulator | pH regulator or acidulant, used in the food and beverage industry to control and adjust the acidity or pH level of products |
| Chelating agent | Controlling metal ions, preventing unwanted reactions, stabilize metals to prevent spoilage, and remove metal impurities from solutions, improving food quality and safety |
| Emulsifying agent | Creation of stable emulsions by stabilizing and blending oil and water-based ingredients, as well as ensuring smooth texture and appearance of food products |
| Food antioxidant | Assissting in preventing oxidation, extending shelf life, and preserving food quality |
| Nutritive sour flavoring agent | Enhacing the sourness or tart flavor of foods and beverages while also offering some nutritional value |
| Nutritive sweetener | Enhancing the sweetness of foods and beverages while also providing calories and energy to food products |
| Preservatives agent | Preventing the spoilage or decay of food products by inhibiting microbial growth |
| Thickening agent | Increasing the viscosity or thickness of food products |
| Water retention agent | Enhancing moisture retention while aslo improving the texture and shelf life of food products |
